# Association Between Neck Circumference and Uncontrolled Hyperglycemia Diabetes in Korean Adults: From the Korea National Health and Nutrition Examination Survey, 2019 to 2021

**DOI:** 10.3390/bioengineering12101099

**Published:** 2025-10-13

**Authors:** Hyeonah Seo, Wonju Yoon, Jae Ho Kim, Byung Chul Shin, Hyun Lee Kim, Minkook Son, Youngmin Yoon

**Affiliations:** 1 Division of Nephrology, Kwangju Christian Hospital, Gwang-ju 61661, Republic of Korea; 2 Department of Neurosurgery, Chosun University Hospital, Chosun University School of Medicine, Gwang-ju 61452, Republic of Korea; jhkim85@chosun.ac.kr; 3 Institute of Well-Aging Medicare & Chosun University G-LAMP Project Group, Chosun University, Gwang-ju 61452, Republic of Korea; 4 Division of Nephrology, Department of Medicine, Chosun University Hospital, Chosun University School of Medicine, Gwang-ju 61452, Republic of Korea; 5 Department of Physiology, Dong-A University College of Medicine, Busan 49315, Republic of Korea; 6 Department of Data Sciences Convergence, Dong-A University College of Medicine, Busan 49315, Republic of Korea

**Keywords:** neck circumference, diabetes mellitus, hyperglycemia, uncontrolled diabetes, Korean National Health and Nutrition Examination Survey

## Abstract

Diabetes mellitus (DM) is a major global health concern, associated with both microvascular and macrovascular complications. Early identification of individuals at risk of hyperglycemia and diabetes progression is crucial for preventing long-term complications and improving patient outcomes. We investigated the association between neck circumference (NC) and hyperglycemia in non-diabetic individuals and in patients with uncontrolled DM, using data from the nationally representative Korea National Health and Nutrition Examination Survey (KNHANES) 2019–2021. Uncontrolled DM was defined as hemoglobin A1c (HbA1c) ≥ 7.0%, while hyperglycemia in non-diabetic individuals was defined as fasting blood glucose ≥ 126 mg/dL or HbA1c ≥ 6.5%. Logistic regression analyses were conducted to evaluate the association between NC and glycemic outcomes. NC was independently associated with hyperglycemia in non-diabetic individuals (Model 1: odds ratio (OR): 1.09; 95% confidence interval (CI): 1.05–1.13; Model 2: OR: 1.09; 95% CI: 1.05–1.13) and patients with uncontrolled DM (Model 1: OR: 1.10; 95% CI: 1.03–1.17; Model 2: OR: 1.11; 95% CI: 1.04–1.18) after adjusting for potential confounders. This study demonstrates that NC is a significant risk factor for hyperglycemia in the general population and for individuals with uncontrolled DM. NC may serve as a simple, non-invasive anthropometric marker to help identify individuals at elevated risk for poor glycemic control.

## 1. Introduction

Diabetes mellitus (DM) is a major global health concern, affecting approximately 537 million individuals worldwide in 2021, accounting for nearly 10.5% of the adult population [1,2,3]. The economic burden of DM is substantial, with global healthcare expenditures totaling USD 966 billion in 2021 and projected to exceed USD 1054 billion by 2045 [1]. This increasing burden is attributed to lifestyle changes, increased obesity rates, urbanization, and aging populations [4,5,6]. DM leads to various microvascular and macrovascular complications, such as diabetic retinopathy, diabetic nephropathy, and cardiovascular diseases (CVDs), all of which significantly impair the quality of life and increase healthcare costs [7,8,9]. Uncontrolled diabetes, characterized by persistent hyperglycemia (glycated hemoglobin [HbA1c] ≥ 7%), significantly increases the risk and progression of diabetes-related complications [10,11]. A previous study showed that a 1% increase in HbA1c above the target level was associated with a 15–20% increased risk of cardiovascular complications and a 25–35% increased risk of microvascular complications [10]. The underlying mechanisms driving these complications include chronic systemic inflammation, oxidative stress, endothelial dysfunction, and insulin resistance, all of which contribute to vascular damage and metabolic dysregulation [12,13]. Early identification of individuals at risk of hyperglycemia and DM progression is critical for preventing long-term complications and improving patient outcomes.

Recently, the relationship between anthropometric measurements and metabolic disorders has been studied [14,15,16]. Traditional measures such as body mass index (BMI), waist circumference (WC), and waist-to-hip ratio have long been established as indicators of metabolic risk [16,17]. Neck circumference (NC) is a novel and promising anthropometric marker of metabolic health. Furthermore, NC is strongly associated with central obesity, metabolic syndrome (MetS), and its components, such as hypertension (HTN), elevated triglycerides, and fasting blood sugar [18,19,20]. Emerging evidence suggests that elevated NC is not only associated with MetS and its components but also strongly correlated with development and poor glycemic control in individuals with diabetes [21,22]. In addition, individuals with higher NC tend to exhibit increased fasting glucose levels, higher HbA1c levels, and reduced insulin sensitivity, highlighting the potential of NC as an early indicator of metabolic dysfunction [21,22]. Unlike BMI and WC, which require precise positioning and measurement protocols, NC can be measured quickly and with high reproducibility, making it an attractive option for large-scale screening and clinical practice. Given its simplicity, ease of measurement, and strong correlation with metabolic risk factors, NC is a practical tool for identifying individuals at risk of diabetes-related complications [23]. However, the relationship between NC and hyperglycemia across different populations, particularly Asian populations, remains to be fully elucidated. In this study, we demonstrated that NC is significantly associated with fasting hyperglycemia, not only in patients with DM but also in normal individuals in the general Korean population.

## 2. Materials and Methods

### 2.1. Study Population

**Figure 1:**
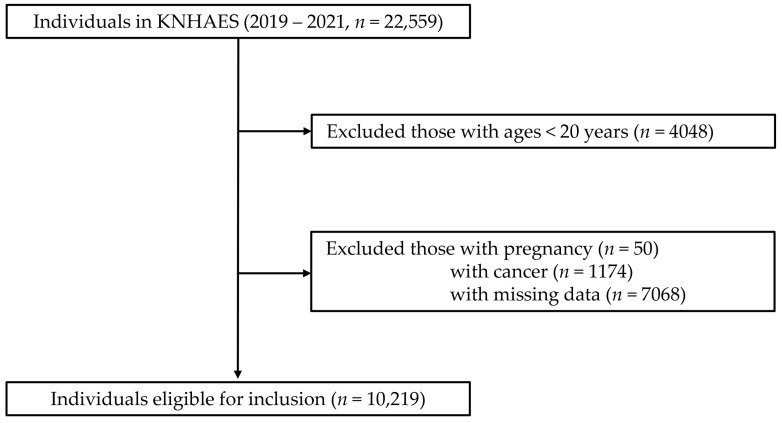
Flow diagram of study population.

This cross-sectional study used data from the Korea National Health and Nutrition Examination Survey (KNHANES) VIII, a nationally representative surveillance system that collects comprehensive health and nutritional data through standardized health interviews, examinations, and nutritional surveys. Data were collected between 2019 and 2021, with an initial enrollment of 22,559 individuals. Individuals younger than 20 years (*n* = 4048), pregnant women (*n* = 50), individuals with a history of cancer (*n* = 1174), and those with missing data (*n* = 7068) were excluded. After exclusion, the final study population consisted of 10,219 individuals (Figure 1).

### 2.2. Demographic Data and Measurements

Trained personnel gathered participant information through questionnaires and one-on-one interviews and collected self-reported demographic information and medical backgrounds, including DM, HTN, and dyslipidemia. Smoking habits were classified into two categories: current smokers and non-smokers; the latter group included former smokers and individuals who had never smoked. Alcohol consumption was also categorized into two groups: non-drinkers, defined as those who had not consumed alcohol in the past year or drank alcohol less than once a month; and drinkers, defined as individuals who consumed alcohol more frequently than once a month. Physical activity levels were divided into two categories: regular exercise, defined as engaging in at least 150 min of moderate-intensity exercise per week, 75 min of high-intensity exercise per week, or an equivalent combination (where one minute of high-intensity exercise equates to two minutes of moderate-intensity exercise); and non-regular exercise, indicating activity levels below these thresholds. The NC measurement protocol required the participants to be properly positioned in a chair, maintaining contact between their back and the chair’s surface, while keeping their head upright and their arms relaxed alongside their body. For male participants, the measurement point was identified at the Adam’s apple, whereas for female participants, measurements were taken at the prominence of the thyroid cartilage. WC measurements were obtained at the intermediate point between the lowest rib margin and the superior border of the iliac crest along the right mid-axillary line. Height and weight measurements followed the standardized KNHANES protocols, and body mass index (BMI) was calculated as weight in kilograms divided by height in meters squared. Blood pressure readings were conducted by qualified nursing staff after participants rested for 5 min in a seated position with proper arm support at heart level. Three separate blood pressure measurements were obtained, including systolic (SBP) and diastolic (DBP) readings. Blood specimens were collected after a minimum of 8 h of fasting at night.

### 2.3. Definition of Uncontrolled DM, Hyperglycemia of Non-DM, and MetS

For this analysis, uncontrolled DM was defined as a hemoglobin A1c (A1c) ≥ 7.0% according to American Diabetes Association (ADA) guidelines [24]. In individuals without DM, hyperglycemia was defined as a fasting blood glucose level ≥ 126 mg/dL or A1c ≥ 6.5% [25]. The diagnosis of MetS was identified in individuals who met at least three of the following five criteria: (1) elevated fasting blood glucose (≥ 100 mg/dL), (2) increased blood pressure (DBP ≥ 85 mmHg or SBP ≥ 130 mmHg), (3) elevated triglyceride levels (TG ≥ 150 mg/dL), (4) reduced HDL cholesterol (< 50 mg/dL in women and < 40 mg/dL in men), and (5) abdominal obesity (WC ≥ 85 cm in women and ≥ 90 cm in men).

### 2.4. Statistical Analysis

**Figure 2:**
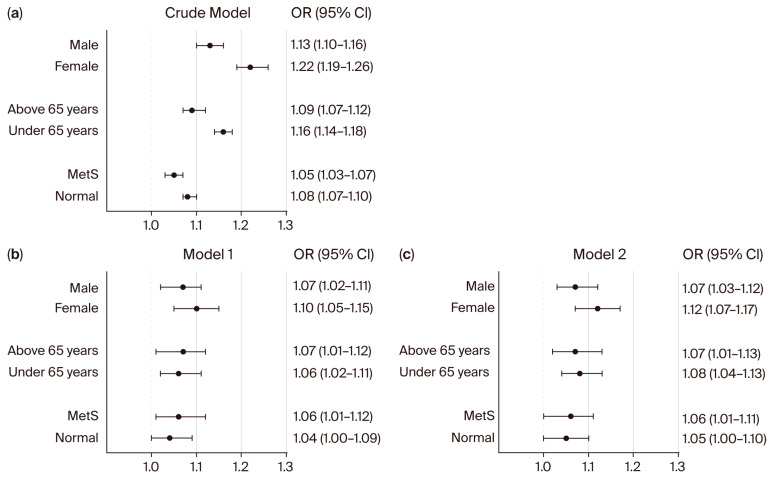
Subgroup analysis for the association between neck circumference and hyperglycemia in non-diabetic individuals. Forest plots show the odds ratios (ORs) for NC and hyperglycemia in univariate (**a**) and multivariate (**b**,**c**) logistic regression analyses. ORs and 95% confidence intervals (CIs) were calculated using univariate and multivariate logistic regression models. Model 1 was adjusted for sex, age, BMI, WC, smoking status, alcohol consumption, regular exercise, HTN, CKD, and dyslipidemia. Model 2 was adjusted for sex, age, BMI, WC, smoking status, alcohol consumption, regular exercise, SBP, DBP, eGFR, and total cholesterol. Abbreviation: MetS: metabolic syndrome.

Continuous variables are expressed as mean ± standard deviation, while categorical variables are presented as frequencies and percentages. Baseline characteristics were analyzed by comparing normal versus hyperglycemic individuals without DM or with controlled DM versus individuals with diagnosed but uncontrolled DM. Univariate and multivariate logistic regression analyses were performed to examine the association between NC and both hyperglycemic individuals and patients with uncontrolled DM, with the results reported as odds ratios (ORs) and 95% confidence intervals (CIs). A multivariate analysis was conducted using two models with progressively adjusted confounders. The crude model is based on a simple model with only one variable. Model 1 was adjusted for sex, age, BMI, WC, smoking status, alcohol consumption, regular exercise, HTN, chronic kidney disease (CKD), and dyslipidemia. Model 2 was further adjusted for sex, age, BMI, WC, smoking status, alcohol consumption, regular exercise, SBP, DBP, estimated glomerular filtration rate, and total cholesterol. Statistical analyses were conducted using SPSS software version 29 (IBM Corporation, Armonk, NY, USA) and Prism version 10.1.2 (GraphPad). Figure 2 was created using Prism version 10.1.2 (GraphPad). Statistical significance was set at *p* < 0.05.

## 3. Results

### 3.1. Baseline Characteristics of the Study Participants

The baseline characteristics of the study participants are summarized in Table 1. A total of 10,219 individuals were included and categorized into a non-DM group (*n* = 8203) and a DM group (*n* = 2016). The non-DM group was further divided into two subgroups: normal glucose (*n* = 5181) and hyperglycemia (*n* = 3022). Hyperglycemia in non-diabetic individuals was defined as a fasting blood glucose level ≥ 126 mg/dL or an A1c ≥ 6.5%. The DM group was stratified into controlled DM (*n* = 1156) and uncontrolled DM (*n* = 860), with uncontrolled DM defined as an HbA1c level ≥ 7.0%.

Males represented a significantly higher proportion in the hyperglycemic subgroup compared to the normal glycemic subgroup (51.2% vs. 37.1%; *p* < 0.01) among non-diabetic participants. Similarly, male predominance was observed in the uncontrolled DM subgroup compared to the controlled DM subgroup (52.8% vs. 52.0%; *p* = 0.72), although this difference was not statistically significant. The hyperglycemic subgroup was significantly older than the normal glycemic subgroup (60.0 ± 11.3 vs. 57.5 ± 11.7 years; *p* < 0.01), while participants with uncontrolled DM were significantly younger than those with controlled DM (63.5 ± 10.6 vs. 65.3 ± 10.4 years; *p* < 0.01). NC showed significant differences across glycemic status groups. Participants with normal glucose levels had the lowest NC (34.3 ± 3.1 cm), whereas hyperglycemic individuals had higher values (35.7 ± 3.3 cm, *p* < 0.01). Among DM patients, NC was greater in the uncontrolled group (36.6 ± 3.4 cm) compared to the controlled group (36.2 ± 3.3 cm, *p* < 0.01).

Triglyceride levels were significantly elevated in both the hyperglycemic and uncontrolled DM subgroups compared to their counterparts (hyperglycemic: 151.6 ± 125.5 vs. normal: 119.6 ± 86.2 mg/dL; uncontrolled DM: 173.0 ± 153.1 vs. controlled DM: 141.1 ± 98.9 mg/dL; both *p* < 0.01). Conversely, HDL cholesterol levels showed an inverse relationship with worsening glycemic status, with the lowest levels observed in the uncontrolled DM subgroup (46.0 ± 11.0 mg/dL). The prevalence of MetS was notably higher in hyperglycemic individuals (50.9%) compared to those in the normal glucose subgroup (12.0%) (*p* < 0.01). Similarly, among DM patients, the uncontrolled group had a higher prevalence of MetS (66.7%) than the controlled group (53.5%) (*p* < 0.01). CKD was also more common in the hyperglycemic group (5.0% vs. 3.2%, *p* < 0.01), although among DM patients, the difference in CKD prevalence between the uncontrolled and controlled groups was not significant (9.0% vs. 10.4%; *p* = 0.29).

### 3.2. Associations of NC and Hyperglycemia and Uncontrolled DM

To validate the association between NC and both hyperglycemia and uncontrolled DM, we conducted univariate and multivariate logistic regression analyses (Table 2). In the crude model, NC showed significant associations with hyperglycemia in non-diabetic individuals (OR: 1.15; 95% CI: 1.14–1.17; *p* < 0.01) and patients with uncontrolled DM (OR: 1.04; 95% CI: 1.02–1.07; *p* < 0.01). In the adjusted models, these associations remained robust. In Model 1, NC was independently associated with hyperglycemia in non-diabetic individuals (OR: 1.09; 95% CI: 1.05–1.13; *p* < 0.01). Among DM patients, NC was also significantly associated with uncontrolled DM (OR: 1.10; 95% CI: 1.03–1.17; *p* < 0.01). Model 2, which incorporated additional adjustments for SBP, DBP, eGFR, and total cholesterol, confirmed the significance of these associations. NC remained significantly associated with hyperglycemia in non-diabetic individuals (OR: 1.09; 95% CI: 1.05–1.13; *p* < 0.01) and those with uncontrolled DM (OR: 1.11; 95% CI: 1.04–1.18; *p* < 0.01). We also evaluated the relationship between NC and DM prevalence (Table 3). The results demonstrated that increased NC was significantly associated with DM prevalence in the crude model (OR: 1.15; 95% CI: 1.13–1.17; *p* < 0.01). This association persisted after adjustments in both Model 1 and Model 2, with NC remaining a significant predictor of DM prevalence (Model 1: OR: 1.19; 95% CI: 1.15–1.24; *p* < 0.01; Model 2: OR: 1.23; 95% CI: 1.18–1.28; *p* < 0.01).

### 3.3. Subgroup Analysis of NC and Both Hyperglycemia and Uncontrolled DM Association

**Figure 3:**
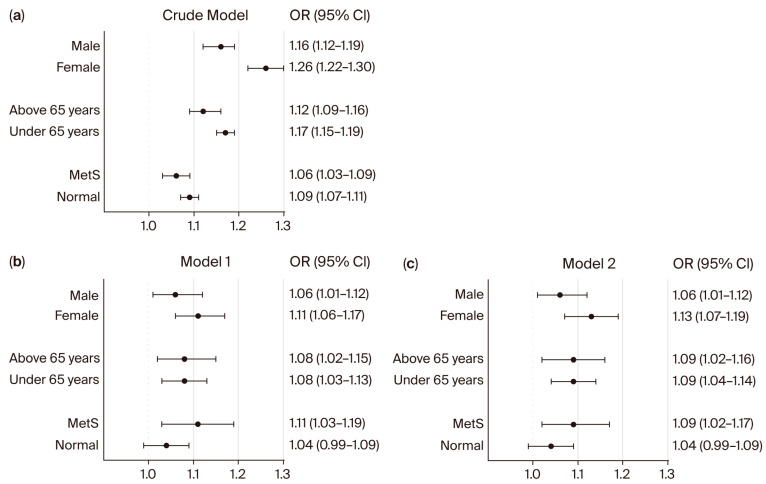
Subgroup analysis for the association between neck circumference and patients with uncontrolled DM. Forest plots show the odds ratios (ORs) for NC and patients with uncontrolled DM in univariate (**a**) and multivariate (**b**,**c**) logistic regression analyses. ORs and 95% confidence intervals (CIs) were calculated using univariate and multivariate logistic regression models. Model 1 was adjusted for sex, age, BMI, WC, smoking status, alcohol consumption, regular exercise, HTN, CKD, and dyslipidemia. Model 2 was adjusted for sex, age, BMI, WC, smoking status, alcohol consumption, regular exercise, SBP, DBP, eGFR, and total cholesterol. Abbreviation: MetS: metabolic syndrome.

We next performed subgroup analyses to evaluate whether the association between NC and hyperglycemia in non-diabetic individuals, as well as those with uncontrolled DM, was consistent across different populations (Figure 2 and Figure 3). In non-diabetic individuals (Figure 2), NC was significantly associated with hyperglycemia in both males and females in the crude model (male: OR 1.13; 95% CI: 1.10–1.16; female: OR 1.22; 95% CI: 1.19–1.26). These associations remained significant after adjustment (Model 2: male: OR 1.07; 95% CI: 1.03–1.12; female: OR 1.12; 95% CI: 1.07–1.17). When stratified by age, the association between NC and hyperglycemia was observed in both younger and older non-diabetic participants in the crude model and remained significant after adjustment for multiple confounders (Figure 2). Among non-diabetic individuals with metabolic syndrome (MetS), NC was significantly associated with hyperglycemia in both crude (OR 1.05; 95% CI: 1.03–1.07) and adjusted models (Model 1: OR 1.06; 95% CI: 1.01–1.12; Model 2: OR 1.06; 95% CI: 1.01–1.11). In contrast, among non-DM individuals without MetS, NC was not significantly associated with hyperglycemia in adjusted models (Model 1: OR 1.04, 95% CI: 1.00–1.09; Model 2: OR 1.05, 95% CI: 1.00–1.10).

In DM patients (Figure 3), NC was also significantly associated with uncontrolled diabetes in both males (OR 1.16; 95% CI: 1.12–1.19) and females (OR 1.26; 95% CI: 1.22–1.30) in the crude model, with persistent associations in adjusted models (Model 2: male: OR 1.06; 95% CI: 1.01–1.12; female: OR 1.13; 95% CI: 1.07–1.19). For age groups among DM patients, significant associations were observed in both patients ≥65 years (crude OR: 1.12; 95% CI: 1.09–1.16; Model 2: OR 1.09; 95% CI: 1.02–1.16) and <65 years (crude OR: 1.17; 95% CI: 1.15–1.19; Model 2: OR 1.09; 95% CI: 1.04–1.14). Among DM patients with MetS, the association between NC and uncontrolled DM remained significant (crude OR: 1.06; 95% CI: 1.03–1.09; Model 1: OR 1.11; 95% CI: 1.03–1.19; Model 2: OR 1.09; 95% CI: 1.02–1.17). However, in DM patients without MetS, NC was not significantly associated with uncontrolled diabetes in adjusted models (Model 1: OR 1.04; 95% CI: 0.99–1.09; Model 2: OR 1.04; 95% CI: 0.99–1.09).

## 4. Discussion

In this study, using a nationwide, cross-sectional, population-based dataset, we demonstrated that NC is a significant risk factor for hyperglycemia in the general population, as well as for participants with uncontrolled DM. The associations remained even after adjusting for several confounding factors, suggesting that NC could serve as an independent anthropometric marker for identifying individuals at risk of metabolic dysregulation.

The global prevalence of DM has significantly increased, posing a major public health burden [26,27,28]. This trend is observed across various regions and demographic groups and is driven by factors such as lifestyle changes, urbanization, aging populations, and dietary transitions [5,28,29]. Maintaining optimal glycemic control is critical for preventing diabetes-related complications [30], including microvascular conditions such as diabetic retinopathy and nephropathy, and macrovascular complications such as CVDs [5,30,31,32].

Glycemic control in individuals with diabetes is typically assessed using fasting glucose and A1c levels [24,33,34]. A1c is a well-established marker of long-term glycemic control, and fasting glucose provides insight into short-term glucose regulation; both require laboratory testing, making them less accessible in resource-limited settings [24,34]. However, the accuracy of HbA1c values can be significantly compromised in patients with anemia, CKD, and vitamin B12 and folate deficiencies [35,36,37,38]. These conditions alter hemoglobin metabolism, leading to misleading HbA1c results due to changes in red blood cell lifespan [35,36]. Additionally, these tests involve blood collection, specialized equipment, and associated costs, which may not be feasible for large-scale screening of certain populations. In contrast, NC is a simple, non-invasive, and cost-effective anthropometric measure that can be easily obtained without the need for laboratory testing. Its strong association with hyperglycemia and metabolic dysfunction suggests that NC could serve as an alternative screening tool for identifying individuals at risk of poor glycemic control, particularly in primary care settings where routine A1c testing may not be available.

Our findings suggest that NC could be a valuable tool for the early identification and management of hyperglycemia, potentially reducing the progression of DM and its associated complications. Previous studies have identified NC as an important anthropometric index associated with obesity, MetS, DM, CVDs, and CKD [20,39,40,41]. In addition, NC has been associated with markers of insulin resistance, such as the Homeostasis Model Assessment of Insulin Resistance (HOMA-IR) [42]. Consistently, our study found that increased NC was associated with DM prevalence in both the crude and adjusted models (Table 3). Baseline characteristics (Table 1) revealed that individuals with hyperglycemia in the non-DM group were older, whereas those with uncontrolled DM in the DM group were relatively younger. Additionally, both hyperglycemic individuals in the non-DM group and those with uncontrolled DM were more likely to be smokers and alcohol drinkers, and had higher BMI and WC compared to individuals with normal glucose levels in the non-DM group and those with controlled diabetes in the DM group. However, despite these confounders, our adjusted models demonstrated that NC remained significantly associated with hyperglycemia in individuals without DM and with those with uncontrolled diabetes. To validate these associations further, we used two adjustment models. Model 1 included categorical confounding variables such as the prevalence of HTN, CKD, and dyslipidemia, whereas Model 2 incorporated continuous confounding variables, including SBP, DBP, eGFR, and total cholesterol. The consistent association between NC and hyperglycemia across both models highlights the potential of NC as an independent anthropometric marker of metabolic risk.

Subgroup analysis revealed that the association between NC and both hyperglycemia in non-diabetic individuals and those with uncontrolled DM was significant only among participants with MetS (Figure 2 and Figure 3). In individuals without MetS, the association between NC and glycemic outcomes was not statistically significant after adjustment, suggesting that the predictive value of NC may be particularly relevant in individuals with an unhealthy metabolic profile. These findings support the hypothesis that NC reflects upper-body adiposity and its associated metabolic burden, which is more pronounced in individuals with MetS. This also underscores the importance of considering overall metabolic context when evaluating the clinical relevance of anthropometric markers such as NC. While traditional anthropometric measures such as BMI and WC remain important tools in clinical assessment, NC has emerged as a promising predictor of metabolic disorders, offering unique insights into upper-body fat distribution [43,44]. This anatomical location is particularly relevant because upper body adiposity plays a crucial role in the pathophysiology of insulin resistance and systemic inflammation [45,46]. The metabolically active fat in this region secretes proinflammatory cytokines such as IL-6 and adipokines, which impair glucose metabolism and elevate cardiovascular risk by promoting hepatic gluconeogenesis, dyslipidemia, and endothelial dysfunction [46,47]. NS is also a recognized risk factor for obstructive sleep apnea, which contributes to insulin resistance and systemic inflammation via intermittent hypoxia-induced oxidative stress and sympathetic activation [48,49].

However, this study had several limitations. First, its cross-sectional design inherently limited its ability to establish causality between NC and uncontrolled DM. While our findings demonstrate strong associations, longitudinal research is necessary to determine whether changes in NC over time can effectively predict the diabetes control status. Second, despite our comprehensive adjustment for confounding variables, the potential influence of unmeasured factors, such as dietary patterns and family history, cannot be completely eliminated. Finally, NC was assessed at a single time point, and variations in NC over time were not examined, which may have affected its long-term predictive value. Despite these limitations, this study had several strengths. One of its key strengths is the use of a large nationwide population-based dataset, which allows for robust subgroup analysis and enhances the generalizability of the findings. Comprehensive adjustments for confounding variables, including BMI, WC, and other metabolic risk factors, further strengthened the validity of the results. Additionally, this study provides valuable insights into the differential associations between NC and hyperglycemia across sex, age, and MetS subgroups.

## 5. Conclusions

Our findings highlight the clinical significance of NC as an independent predictor of hyperglycemia in individuals without DM and those with uncontrolled diabetes. Given its strong association with metabolic risk factors, NC has the potential to be a simple yet effective screening tool for identifying individuals at an increased risk of glycemic dysregulation and diabetes. Future research should focus on longitudinal studies to establish a temporal relationship between changes in NC and metabolic outcomes. Additionally, further investigations of population-specific NC thresholds and the potential impact of targeted interventions aimed at reducing neck adiposity may enhance its clinical utility. The integration of NC measurements into routine clinical practice may improve early detection and risk stratification, ultimately contributing to improved diabetes prevention and management strategies.

## Figures and Tables

**Table 1:** Baseline characteristics of study population.

Variables	Non-DM (*n* = 8203)			DM (*n* = 2016)		
Study Population (*n* = 10,219)	Normal (*n* = 5181)	Hyperglycemic (*n* = 3022)	*p*-Value	Controlled (*n* = 1156)	Uncontrolled (*n* = 860)	*p*-Value
Sex (%)						
Male	1922 (37.1)	1546 (51.2)	<0.01	601 (52.0)	454 (52.8)	0.72
Female	3259 (62.9)	1476 (48.8)		555 (48.0)	406 (47.2)	
Age (years)	57.5 ± 11.7	60.0 ± 11.3	<0.01	65.3 ± 10.4	63.5 ± 10.6	<0.01
Blood analysis						
Hemoglobin (g/dL)	13.6 ± 1.5	14.1 ± 1.5	<0.01	13.8 ± 1.6	14.0 ± 1.6	<0.01
Glucose (mg/dL)	91.8 ± 5.3	108.1 ± 14.0	<0.01	119.9 ± 18.1	154.1 ± 44.1	<0.01
Hemoglobin A1c (%)	5.6 ± 0.3	5.8 ± 0.4	<0.01	6.4 ± 0.4	8.1 ± 1.3	<0.01
Total cholesterol (mg/dL)	196.7 ± 37.3	196.6 ± 36.5	0.44	172.4 ± 41.6	171.6 ± 45.2	0.69
HDL cholesterol (mg/dL)	53.8 ± 13.1	50.4 ± 12.1	<0.01	47.9 ± 11.5	46.0 ± 11.0	<0.01
Triglyceride (mg/dL)	119.6 ± 86.2	151.6 ± 125.5	<0.01	141.1 ± 98.9	173.0 ± 153.1	<0.01
eGFR	89.3 ± 18.2	87.7 ± 17.8	<0.01	83.6 ± 20.1	86.8 ± 22.0	<0.01
Blood pressure						
Systolic blood pressure (SBP; mmHg)	119.8 ± 16.9	125.2 ± 15.7	<0.01	127.0 ± 16.4	126.9 ± 15.7	0.86
Diastolic blood pressure (DBP; mmHg)	75.5 ± 9.7	77.8 ± 9.8	<0.01	74.6 ± 9.6	75.6 ± 10.3	0.03
Body measurements						
Body mass index (BMI; kg/m^2^)	23.5 ± 3.1	24.8 ± 3.2	<0.01	25.3 ± 3.5	25.6 ± 3.8	0.06
Waist circumference (WC; cm)	82.6 ± 9.3	87.3 ± 9.0	<0.01	89.8 ± 9.3	91.1 ± 9.6	<0.01
Neck circumference (NC; cm)	34.3 ± 3.1	35.7 ± 3.3	<0.01	36.2 ± 3.3	36.6 ± 3.4	<0.01
Health interview (**%**)						
Current smoker	629 (12.1)	441 (14.6)	<0.01	165 (14.3)	175 (20.3)	<0.01
Alcohol consumption	1862 (35.9)	1386 (45.9)	<0.01	420 (36.3)	319 (37.1)	0.72
Regular exercise	2079 (40.1)	1152 (38.1)	0.07	407 (35.2)	310 (36.0)	0.70
Underlying diseases (%)						
Hypertension (HTN)	1637 (31.6)	1431 (47.4)	<0.01	763 (66.0)	498 (57.9)	<0.01
Dyslipidemia	2167 (41.8)	1664 (55.1)	<0.01	801 (69.3)	640 (74.4)	0.02
Chronic kidney disease (CKD)	168 (3.2)	152 (5.0)	<0.01	120 (10.4)	77 (9.0)	0.29
Metabolic syndrome (MetS)	622 (12.0)	1537 (50.9)	<0.01	618 (53.5)	574 (66.7)	<0.01

Values for continuous variables are presented as mean ± standard deviation. *p*-values for sex, current smoking status, alcohol consumption, regular exercise, HTN, dyslipidemia, CKD, and MetS were calculated using the Chi-squared test. *p*-values for hemoglobin, glucose, total cholesterol, HDL cholesterol, triglyceride, eGFR, BMI, WC and NC were calculated using Student’s *t*-test.

**Table 2:** Association between neck circumference and hyperglycemia and uncontrolled DM.

Neck Circumference	Crude Model		Adjusted Model			
			Model 1 ^a^		Model 2 ^b^	
	OR (95% CI)	*p*-Value	OR (95% CI)	*p*-Value	OR (95% CI)	*p*-Value
Non-diabetic patients	1.15	<0.01	1.09	<0.01	1.09	<0.01
	(1.14–1.17)		(1.05–1.13)		(1.05–1.13)	
DM patients	1.04	<0.01	1.10	<0.01	1.11	<0.01
	(1.02–1.07)		(1.03–1.17)		(1.04–1.18)	

^a^ Model adjusted for sex, age, BMI, WC, current smoking status, alcohol consumption, regular exercise, HTN, CKD, and dyslipidemia. ^b^ Model adjusted for sex, age, BMI, WC, current smoking status, alcohol consumption, regular exercise, SBP, DBP, eGFR, and total cholesterol.

**Table 3:** Association between neck circumference and DM.

Neck Circumference	Crude Model		Adjusted Model			
			Model 1 ^a^		Model 2 ^b^	
	OR (95% CI)	*p*-Value	OR (95% CI)	*p*-Value	OR (95% CI)	*p*-Value
All subjects	1.15	<0.01	1.19	<0.01	1.23	<0.01
	(1.13–1.17)		(1.15–1.24)		(1.18–1.28)	

^a^ Model adjusted for sex, age, BMI, WC, current smoking status, alcohol consumption, regular exercise, HTN, CKD, and dyslipidemia. ^b^ Model adjusted for sex, age, BMI, WC, current smoking status, alcohol consumption, regular exercise, SBP, DBP, eGFR, and total cholesterol.

## Data Availability

The information was obtained from the Korea National Health and Nutrition Examination Survey (KNHANES), organized by the Korea Centers for Disease Control and Prevention (KCDCP), and can be freely obtained from the KCDCP website (https://knhanes.cdc.go.kr accessed on 29 March 2025).

## Abbreviations

The following abbreviations are used in this manuscript:

DM	Diabetes mellitus
CVD	Cardiovascular disease
BMI	Body mass index
WC	Waist circumference
NC	Neck circumference
MetS	Metabolic syndrome
CKD	Chronic kidney disease
HTN	Hypertension
